# The Effect of Resin-modified Glass Ionomer Containing Bacterial Cellulose Nanocrystal as a Base on the Fracture Resistance of Class II Restorations

**DOI:** 10.4317/jced.63458

**Published:** 2025-12-30

**Authors:** Marzie Moradian, Maryam Saadat, Milad Khoubani

**Affiliations:** 1 Department of Operative dentistry, School of Dentistry, Shiraz University of Medical Science, Shiraz, Iran; 2 Student Research Committee, School of Dentistry, Shiraz University of Medical Science, Shiraz, Iran

## Abstract

**Background:**

Resin-modified glass ionomer cements (RMGICs) are commonly used as base materials in restorative dentistry due to their fluoride release and chemical bonding properties. Incorporating bacterial cellulose nanocrystals (BCNCs) into RMGIC may improve its mechanical properties, especially fracture resistance (FR), which is critical in Class II restorations. This study aimed to evaluate the effect of BCNC-reinforced RMGIC on the fracture resistance and fracture patterns mesio-occluso-distal (MOD) cavities restored premolars using the sandwich technique.

**Material and Methods:**

Sixty sound human maxillary premolars were randomly divided into five groups (n = 12) based on the restoration protocol: Group I) intact teeth (positive control); Group II) MOD cavities left unrestored (negative control); Group III) MOD cavities restored with short-fiber reinforced composite (SFRC) only; Group IV) MOD cavities restored with conventional RMGIC + SFRC; Group V) MOD cavities restored with 1% BCNC-reinforced RMGIC + SFRC. The restorations in groups III to V were finally covered with 1 mm of nanohybride composite. All specimens underwent thermocycling. Fracture resistance was tested using a universal testing machine, and fracture patterns were classified as restorable or unrestorable based on the CEJ level. Data were analyzed using one-way ANOVA and Tukey's post-hoc test (P &lt; 0.05).

**Results:**

The negative control group (Group II) exhibited significantly lower fracture resistance compared to all other groups (P &lt; 0.001). Group V (BCNC-modified RMGIC+SFRC) showed the highest fracture resistance, but no significant differences were found among Groups I (intact teeth), III (SFRC only), IV (RMGIC+SFRC), and V (P &gt; 0.05). Group II had predominantly unrestorable fractures, while other groups showed a higher proportion of restorable fractures.

**Conclusions:**

The incorporation of BCNC into RMGIC as a base in Class II restorations improved fracture resistance and resulted in more favorable fracture patterns, suggesting its potential as a reinforcing agent in restorative dentistry.

## Introduction

Posterior teeth are routinely subjected to various factors that compromise their structural integrity and fracture resistance (1). These include long-standing dental caries, traumatic injuries, non-carious cervical lesions, extensive cavity preparations, and root canal treatments (2). Class II mesio-occluso-distal (MOD) cavities involving both proximal surfaces pose significant clinical challenges due to extensive structural loss (3 , 4). A range of direct and indirect restorative approaches, such as amalgam, resin composites, and full-coverage restorations, are utilized to restore such teeth, each with specific limitations and clinical considerations (5). All these treatment modalities aim to reinforce the weakened tooth structure and reduce fracture risk (6 - 8). Alternative techniques have been proposed to address the limitations associated with direct composite restorations in Class II cavities. One such approach is the open sandwich technique, which involves the placement of a glass ionomer cement (GIC) base beneath the composite restoration (9). GICs, due to their lower elastic modulus, may better absorb occlusal stresses and reduce the risk of catastrophic failure (10). Previous studies have shown that the use of conventional GICs as a base layer can enhance the fracture resistance of restored teeth, sometimes achieving values comparable to intact teeth. Furthermore, the development of resin-modified glass ionomer cements (RMGICs) has improved handling characteristics and working time by allowing light-curing of the material (11 - 14). In recent years, nanotechnology has introduced bacterial cellulose nanocrystals (BCNCs) as promising additives to improve the mechanical performance of dental materials. Studies have demonstrated that BCNCs can significantly increase compressive strength, diametral tensile strength, and the elastic modulus of dental composites and GICs by enhancing the matrix continuity, increasing crosslinking density, and improving stress distribution (15 - 17). Moradian et al. reported that the incorporation of 1 wt% BCNC into RMGIC resulted in enhanced mechanical properties and improved shear bond strength to the dentin, suggesting that BCNC acts as an effective reinforcing nanofiller through a matrix reinfoecement mechanism (15 , 18 , 19). Similarly, Mohammadi et al. found that BCNC incorporation into conventional GIC significantly increased the micro-shear bond strength to the primary tooth dentin (16). In parallel, short-fiber reinforced composites (SFRCs) have gained attention for their superior fracture toughness compared to conventional particulate-filled composites (PFCs). The inclusion of short fibers improves resistance to crack propagation and distributes stress more evenly, thereby enhancing the durability of restorations (20 , 21). Despite these advances, no data currently exists on the effect of BCNC-reinforced RMGIC as a base on the fracture resistance of MOD Class II cavities restored with SFRCs; hence, this study was designed to investigate this effect, under the null hypothesis that the incorporation of BCNC into RMGIC will not significantly influence the fracture resistance of such restorations.

## Material and Methods

**Figure 1:**
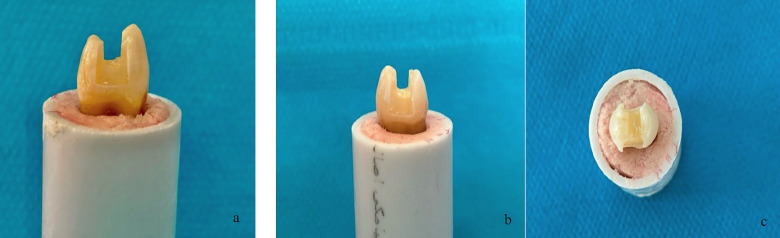
Standard MOD tooth preparation. (A) and (b) medial and distal views; (c) occlusal view.

-Specimens selection The study protocol was reviewed and approved by the Research Ethics Committee of Shiraz University, approval with the code of IR.SUMS.DENTL.REC.1403.044. In this in-vitro experimental study, sixty sound human maxillary premolars extracted for orthodontic purposes were collected from patients aged 18-30 years. Teeth with enamel cracks, fractures, restorations, developmental defects, erosion, or attrition were excluded. After debridement of soft tissues and calculus, specimens were stored in 2% Chloramine-T solution at 5°C for up to three months. Each tooth was mounted in cylindrical acrylic resin blocks (15 mm diameter, 25 mm height) using Teflon molds, with the resin level 2 mm apical to the cementoenamel junction (CEJ). -Preparation of specimens Standardized mesio-occluso-distal (MOD) cavities were prepared using a flat-end cylindrical diamond bur (Jota, Switzerland) mounted on a high-speed handpiece with water cooling (Fig. 1).

1

**Figure 2:**
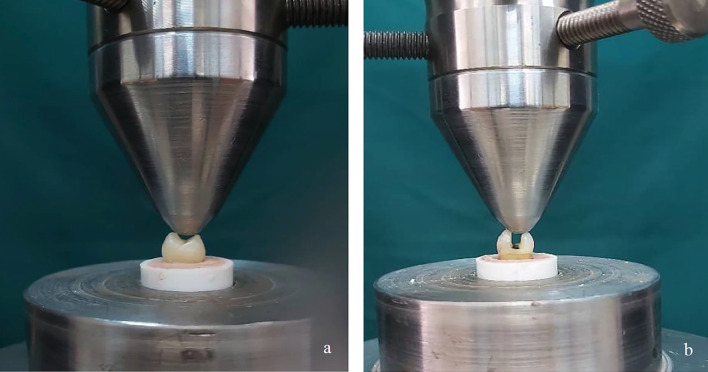
a,b) fracture resistance test by Universal testing machine.

All cavity preparations were performed by a single blinded operator to minimize variability and ensure standardization. The standardized dimensions were occlusal width = half the intercuspal distance, pulpal depth = 2 mm, proximal box width = half the faciolingual dimension, axial wall depth = 1.5 mm, and occluso-gingival height = 4 mm with gingival margins placed above the CEJ. -Restoration procedure The specimens were randomly divided into five groups (n=12): Group I (Positive Control): No cavities prepared. Group II (Negative Control): MOD cavity preparations were completed but left unrestored. Group III: MOD cavities restored with short-fiber reinforced composite (SFRC; everX flow, GC Corporation, Tokyo, Japan). Group IV: MOD cavities restored with a resin-modified glass ionomer cement (RMGIC; Fuji II LC, GC Corporation, Tokyo, Japan) and SFRC composite restoration. Group V: MOD cavities restored with RMGIC containing 1% bacterial cellulose nanocrystals (BCNC; Nano Novin Polymer Co., Iran) and SFRC composite restoration. The cavities were etched with 37% phosphoric acid for 15 seconds, rinsed, and gently dried. Adper Single Bond 2 (3M ESPE, St. Paul, MN, USA) was applied in two consecutive layers, gently dried, and light-cured for 10 seconds, using a halogen curing unit (Monitex GT-1200, Taipei, Taiwan) at 1200 mW/cm² intensity. SFRC composite restorations were performed using the Tofflemire matrix system and light-cured for 40 seconds. For SFRC restorations, the 1-mm occlusal part of the cavity was capped with Z250 composite (3M ESPE, St. Paul, MN, USA). In Groups IV and V, RMGIC was applied before bonding: Group IV: A thin (approximately 1 mm) RMGIC was applied to the gingival walls and light-cured for 20 seconds. Group V: The RMGIC was modified with 1% BCNC. Commercially available BCNC powder (Nano Novin Polymer Co., Iran) was used. To prepare the modified RMGIC, 1 wt% BCNC was manually blended with the RMGIC powder for 30 seconds using a spatula. The mixture was then transferred into an empty amalgam capsule and mixed using an amalgamator (Ultramat 2, SDI, Australia) for 10 seconds to ensure homogenization. Finally, the BCNC-modified powder was combined with the RMGIC liquid on a glass slab using the manufacturer's recommended powder-to-liquid ratio (3.2:1) and the mixture was light-cured for 20 seconds. All specimens were stored in distilled water at 37°C for 24 hours, then thermocycled 1000 cycles between 5°C and 55°C, with a dwell time of 30 s and 10 sec transfer time, using a thermocycling machine (Nemo Technologics, Iran) to simulate oral aging. -Fracture resistance test Each specimen was embedded in self-cure acrylic resin (Acropars, Iran) up to 1 mm below the CEJ. A compressive load was applied using a universal testing machine (Zwick/Roell, Germany) with a 5 mm stainless steel cylindrical rod positioned at the central fossa along the long axis of each tooth. Specimens were subjected to vertical compression with a maximum force of 5 kN and a crosshead speed of 1 mm/min until fracture occurred (Fig. 2).

2 3

**Figure 3:**
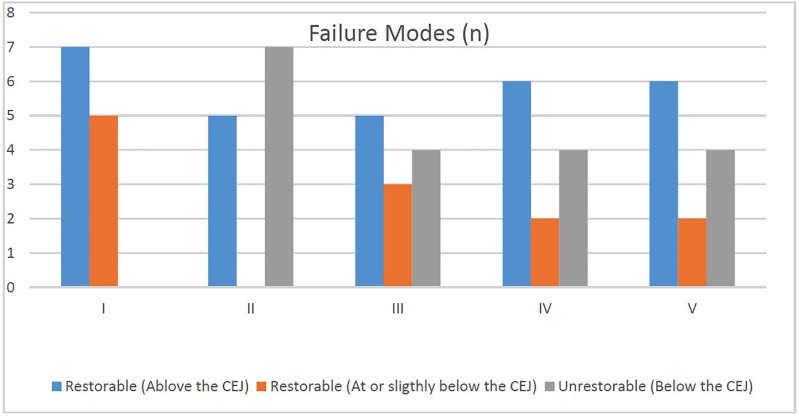
Frequencies (n) for comparison between failure modes of different groups.

The maximum load at fracture (N) was recorded for each sample. The results were recorded using NEXYGEN Plus 3 Data Analysis Software. -Statistical analysis Normality of fracture resistance data was verified using Shapiro-Wilk tests (p &gt; 0.05). The data were transformed using the natural logarithm (Ln_FR) to ensure normality and homogeneity of the variance. Transformed values were analyzed using one-way ANOVA to evaluate the differences among the five groups. The Tukey HSD post-hoc multiple comparison test was used to determine specific group differences, with statistical significance set at p &lt;0.05.

## Results

-Fracture resistance analysis (Ln_FR) The fracture resistance values, expressed as the natural logarithm (Ln_FR), demonstrated statistically significant differences among the five experimental groups (ANOVA: F (4,55) = 8.225, p &lt; 0.001) (Table 1).

Table 1

Tukey's post hoc test revealed Group II (unrestored MOD cavities) exhibited the lowest mean fracture resistance, significantly lower than all other groups (p &lt; 0.05). However, there were no statistically significant difference in mean fracture resistance between the intact teeth (group I) and the three restored groups with, namely SFRC (Group III), RMGIC + SFRC (Group IV), and BCNC-modified RMGIC + SFRC (Group V) (p &gt; 0.05 for all comparisons with Group I). These results indicate that all three restoration techniques were able to restore the fracture resistance lost due to cavity preparation to a level statistically comparable to sound, unprepared teeth, with Group V showing the highest numerical mean value among the restored groups. -Fracture location and restorability The distribution of fracture heights and restorability status across the five groups is summarized in Figure 3.

4

**Figure 4:**
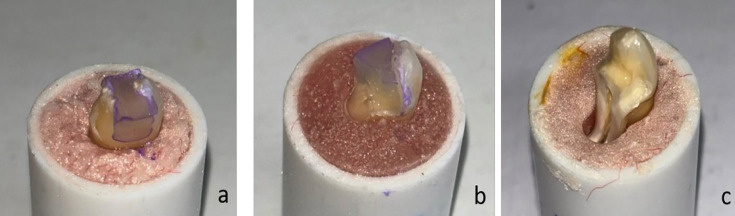
A representative photograph showing: (a) fracture located above the CEJ, (b): fracture occurring at the CEJ level, (c): fracture located below the CEJ.

The fracture heights for each group are categorized as: Over cementoenamel junction (CEJ): Fractures located above the CEJ (Fig. 4a).

At the height of CEJ: Fractures occurring exactly at the CEJ level (Fig. 4b). Under CEJ: Fractures below the CEJ, typically associated with unrestorable damage (Fig. 4c). Group I (intact teeth) and Group V (BCNC-modified RMGIC + SFRC) had the highest proportion of favorable (restorable) fractures above the CEJ. In contrast, Group II (unrestored MOD) showed the greatest number of non-restorable fractures, with a majority of fracture lines located below the CEJ.

## Discussion

Premolars were selected as the tooth model for this study due to their unique anatomical and functional characteristics, which present significant challenges for restorative procedures. Unlike molars, premolars must withstand substantial occlusal forces while also fulfilling aesthetic demands due to their partial visibility in the smile zone (16 , 22). Their smaller size, thinner dentinal walls, and more intricate occlusal morphology further complicate restoration, making them ideal candidates for testing the efficacy of advanced restorative materials and techniques (22 , 23). Class II MOD cavity preparations have been shown to substantially weaken the structural integrity of the teeth by removing key components such as marginal ridges and pericervical dentin. This structural loss can reduce fracture resistance by up to 60% compared to intact teeth (20 , 24). In the present study, the lowest fracture resistance values were observed in Group II (unrestored control), which reinforces these findings and underscores the need for restorative intervention to restore mechanical stability and prevent catastrophic fractures. The high incidence of unrestorable fractures in this group, particularly those extending below the CEJ, highlights the importance of materials that not only restore strength but also redirect or mitigate stress propagation within the tooth-restoration complex (25). In contrast, Groups III to V, which received various restorative combinations, demonstrated significantly improved fracture resistance. Group V (BCNC-modified RMGIC + SFRC) showed the highest mean fracture resistance although the difference was not statistically significant when compared to Group III (SFRC) and Group IV (RMGIC + SFRC). This finding suggests that while BCNCs may not dramatically shift numerical outcomes in small sample sizes, their inclusion contributes meaningfully to overall structural reinforcement and stress redistribution, potentially enhancing clinical outcomes over time. The superior performance of Group V can be attributed to the synergistic effect of bacterial cellulose nanocrystals (BCNC) in the base material and short fibers in the overlaying composite. BCNCs are known to form a dense hydrogen-bond network with polyacrylic acid and glass particles, reinforcing the ionomer matrix and improving the bond to the dentin through chemical interaction (16 , 18). This chemical enhancement complements the micromechanical retention offered by RMGIC and improves the sealing ability and durability of the base layer. Short fiber-reinforced composites (SFRCs) in the occlusal portion further bolster the restoration by acting as a stress-absorbing layer. The embedded E-glass fibers within the SFRC matrix deflect and arrest crack propagation, enhancing resistance to compressive and tensile forces (20 , 26). Additionally, the random orientation of short fibers facilitates multidirectional stress distribution, which is particularly valuable in high-load-bearing premolars. The ability of SFRC to act as an elastic stress breaker between rigid dentin and brittle restorative materials may also explain its consistent performance across Groups III-V. Group IV, which combined conventional RMGIC and SFRC, also showed favorable results, highlighting the importance of a hybrid layering approach that integrates bioactive bases with fiber reinforcement. However, the slightly lower performance compared to Group V suggests that BCNCs provide an additional advantage in fortifying the RMGIC matrix, especially in resisting marginal degradation and microleakage under thermomechanical loading (15 , 18 , 19). The improved performance in the restored groups was further validated under thermocycling, a testing method that replicates intraoral thermal stress. Thermocycling not only simulates aging but also reveals vulnerabilities at the tooth-restoration interface due to thermal expansion mismatch or degradation of the adhesive interface (27 , 28). The durability of Groups III-V under such conditions reinforces the suitability of these materials for long-term application. Despite the positive trends observed, the lack of statistical significance among the top-performing groups may be due to the limited sample size of the study (n = 12 per group). Given the small intergroup differences and biological variability in natural teeth, larger sample sizes are needed to confirm subtle yet clinically relevant differences. Furthermore, the in-vitro nature of the study inherently limits generalization to clinical settings. Variables such as occlusal dynamics, salivary enzymes, and patient-specific factors could influence long-term outcomes. Nonetheless, the data suggest that incorporating BCNCs into RMGIC, followed by a fiber-reinforced composite overlay, provides a promising restorative strategy for structurally compromised premolars. This layered biomimetic approach addresses critical restorative goals,such as resistance to fracture, stress modulation, marginal integrity, and potential caries inhibition,making it highly relevant for clinical translation in high-stress posterior restorations (29 , 30). This study has several limitations, including its in-vitro design, the potential influence of variations in tooth morphology and cavity preparation and the absence of advanced interfacial characterization techniques such as FTIR or XRD to verify the homogeneous dispersion of BCNC within the RMGIC matrix. Future work should include clinical trials, and advanced analytical methods to confirm and extend the present findings.

## Conclusions

The results of this study demonstrated that the addition of bacterial cellulose nanocrystals (BCNC) to resin-modified glass ionomer cement (RMGIC), combined with short fiber-reinforced composite (SFRC) in the open sandwich technique, offers promising benefits for enhancing fracture resistance in Class II MOD cavities in premolars. BCNC reinforcement may contribute to improved mechanical performance and stress distribution within the tooth-restoration system. Therefore, this study provides a foundation for advancing biomimetic restorative techniques to enhance both mechanical and clinical outcomes.

## Figures and Tables

**Table 1:** Mean (±SD) of the natural logarithm of fracture resistance (Ln_FR).

Group	Restoration Type	Mean Ln_FR	Standard Deviation
I	Positive Control (intact teeth)	7.1300A	0.2978
II	Negative Control (unrestored cavities)	6.4577B	0.3612
III	SFRC only	6.8628A	0.3705
IV	RMGIC + SFRC	6.9632A	0.3984
V	BCNC-modified RMGIC + SFRC	7.1759A	0.2894

Means with different superscript letters indicate statistically significant differences (p< 0.05).

## Data Availability

The data used and/or analyzed during the current study are available from the corresponding author on reasonable request.
